# Short report: Performance evaluation of the Idylla™ *KRAS* and *EGFR* mutation tests on paraffin-embedded cytological NSCLC samples

**DOI:** 10.1186/s13000-021-01121-3

**Published:** 2021-08-03

**Authors:** Saskia Offerman, Clemens F. Prinsen, Ageeth Knol, Natalie Methorst, Jeanette Kamphorst, Maarten Niemantsverdriet

**Affiliations:** 1 Isala Pathology, Dr. Van Heesweg 2, 8025 AB Zwolle, Postbus 10400, 8000 GK Zwolle, The Netherlands; 2 Department Pathology C66, Canisius Wilhelmina Ziekenhuis, Weg door Jonkerbos 100, 6532 SZ Nijmegen, The Netherlands

## Abstract

**Background:**

Quick and reliable testing of *EGFR* and *KRAS* is needed in non-small cell lung cancer (*NSCLC)* to ensure optimal decision-making for targeted therapy. The Idylla™ platform was designed for Formalin-Fixed Paraffin-Embedded (FFPE) tissue sections but recently several studies were published that evaluated its potential for cytological specimens. This study aimed to validate the Idylla™ platform for the detection of EGFR/KRAS mutations in cytological *NSCLC* samples prepared as cytoblocks using AGAR and paraffin embedding.

**Material and methods:**

The *KRAS* Idylla™ test were performed on 11 specimens with a known *KRAS* mutation. The *EGFR* Idylla™ test was performed on 18 specimens with a known primary *EGFR* mutation and 7 specimens with a primary EGFR-EGFR T790M resistance mutation combination.

**Results:**

Concordant *KRAS and primary EGFR* mutations were detected for both *KRAS* and primary *EGFR* mutations. Samples with a total CQ value of < 26 could be considered negative. Samples with a total CQ value of > 26 could not be assessed (probability of false-negative). In specimens with a primary *EGFR*-*EGFR* T790M resistance mutation combination, 5/7 cases were not concordant.

**Conclusion:**

Our results confirm the conclusion of recent reports that the Idylla™EGFR assay is not suitable in a resistance to EGFR TKI setting, also not in our cytological NSCLC samples prepared as cytoblocks using AGAR and paraffin embedding. *KRAS* and primary *EGFR* mutations were detected using the Idylla™ assays in virtually all cytological NSCLC samples. This analysis was rapid and time-saving compared to other mutation detection assays and may be useful if the amount of material is insufficient to perform a full set of molecular tests.

## Introduction

Eligibility for targeted therapy with specific Tyrosine Kinase Inhibitors (TKI’s) for non-Small Cell Lung Cancer (NSCLC) is assessed by analysis of tumor tissue of either the primary tumor or mediastinal lymph node metastasis for specific mutations [1–3]. However, a diagnosis is often made on cytological specimens because tissue biopsies are not always possible to obtain. Also, for patient comfort it is favored to collect cytological material as this method is considered minimally invasive [1–3]. Mutation analysis on cytological samples of paraffin-embedded cytological specimens of NSCLC patients obtained with endoscopic-ultrasound-guided fine needle aspiration (EUS) and endobronchial-ultrasound–guided fine-needle aspiration (EBUS) transbronchial and transesophageal aspirates has been previously reported [4].

In patients with advanced or metastatic NSCLC targeted treatment with first or second- generation *EGFR* TKI’s can be given after detection of activating *EGFR* mutations [1–3]. Approximately 15–40% of NSCLC adenocarcinoma patients harbor activating *EGFR* mutations [1–3]. Resistance to *EGFR* therapy eventually occurs in all patients treated with first or second-generation *EGFR* TKI’s. In approximately half of these patients, resistance is associated with a secondary mutation, the T790M mutation in *EGFR*, which can be treated with the third-generation *EGFR* TKI osimertinib [1–3]. The T790M mutation is particularly hard to detect in cytological samples since the number of cells obtained after TKI resistance is often low and the T790M mutation is usually present in only a low percentage of the tumor cells [1–4]. The *BRAF* p.(V600E) mutation can be detected in approximately 3% of NSCLC adenocarcinomas [3,5,6]. BRAF targeted therapy has been introduced for the treatment of *BRAF* p.(V600E)-mutated NSCLC [3,5,6]. *KRAS* activating mutations are found in 25–30% of non-small cell lung cancer (NSCLC) samples and these samples rarely harbor other targetable driver mutations [1].

To detect mutations in hotspots of genes like *EGFR* and *KRAS*, one can use a variety of sequencing techniques (e.g. Sanger, NGS) that can detect single or multiple targets [1,2]. These techniques are often elaborate, require highly trained personnel and take up to a week to complete [1,7].

The real-time PCR-based Idylla™ (Biocartis, Belgium) platform uses a disposable cartridge and, offers a fully automated, easy, and fast way of molecular testing of the main hotspots of genes including *EGFR* and *KRAS.* A gene-specific cartridge is available for each of these genes that detects common mutations in hotspot areas [7–11]. Formaldehyde-fixed paraffin-embedded (FFPE) tissue sections are inserted into an assay cartridge that has all required reagents on board including primers and probes, and results are available within a few hours. This system has been used successfully for the detection of *KRAS, EGFR,* and/or *BRAF* mutations on routine FFPE tissue of colorectal carcinoma, melanoma, lung cancer, thyroid cancer, and breast cancer [7–11] and stained cytological NSCLC smears [12].

The Idylla™ platform was developed and CE-IVD approved for FFPE tissue samples but it had not been validated extensively for paraffin-embedded (lung) cytology samples until recently. Recent reports suggest that the Idylla assay is not suitable for the assessment of cytological FFPE samples in the setting of EGFR TKI-resistance [13, 14] This study aimed to evaluate the Idylla™ platform for cytological NSCLC samples prepared as cytoblocks using AGAR and paraffin embedding.

## Materials and methods

### Sample preparation

Pleural fluid, EUS, and EBUS cytological NSCLC samples processed comparably to FFPE tissue samples were obtained. Pleural fluid, or EUS/EBUS aspirations per site (3–4 passes in different directions of the tumor or enlarged mediastinal lymph node of NSCLC patients) were performed and deposited in carbowax 2% fixative. Cell blocks were made using cell pellets embedded in AGAR 10%, followed by formalin fixation, dehydration, and paraffinization as described [4]. All samples were harvested between 2015 and 2018 and analyzed in the last three months of 2018 or the first three months of 2019. For all samples, tumor cell percentage was estimated and specimens were analyzed during routine diagnostics for *KRAS*, *EGFR,* or *BRAF* hotspot mutations during routine molecular diagnostics procedure with high resolution melting (HRM) PCR and reflex Sanger sequencing in our ISO15189 certified lab as described [11]. EGFR-TKI resistance cases were additionally analyzed with NGS because variant allele frequency (VAF) cannot be quantified with Sanger sequencing and the EGFR T790M is usually found in only a fraction of tumor cells with the primary EGFR mutation [1–3].

### Study design

Based on the known *KRAS*, *EGFR,* or *BRAF* status, specimens were selected to be tested with the Idylla™ KRAS Mutation Test (CE/IVD), Idylla™ EGFR Mutation Test (CE/IVD), and/or the Idylla™ BRAF Mutation Test (CE/IVD). The Idylla™ KRAS Mutation Test is solely IVD validated for the use of FFPE human colorectal tissue samples and the Idylla™ EGFR Mutation Test is only IVD validated for the use of FFPE human NSCLC baseline tissue samples. In this research study, we investigate the use of both tests outside its intended use, being human FFPE cytological NSCLC samples. Cytological samples were prepared as cytoblocks using AGAR before paraffin embedding. The *KRAS* Idylla™ test was performed on 11 cytological specimens of NSCLC cases with a known *KRAS* mutation. The *EGFR* Idylla™ test was performed on 18 cytological NSCLC specimens with a known primary *EGFR* mutation only and on 7 cytological specimens of NSCLC cases with two known *EGFR* mutations including the T790M resistance mutation. Idylla samples were analyzed using the Idylla.

Explore analysis software (V.3.2).

### Analysis

After routine diagnostics 10 additional slides were cut in serial sections. HE stainings were performed on the first and last slides and tumor cell percentage was estimated by the same pathologist for all samples used in this study. Because T790M VAF in Idylla™ samples could not be measured exactly it was estimated using the formula (TC% Idylla™/TC% original sample)*T790M% original sample. 1–4 slides (4 μm) were used per cartridge and analyzed following the procedure as described in the Instructions for Use (IFU) of the corresponding Idylla™ test. The Idylla™ Explore software was used to obtain the CQ values of the qPCR curves. Cases were usually first analyzed with 1 slide of material using Idylla™ *EGFR* cartridges (Table 2.). When the mutation was not detected using 1 slide, more slides were used in subsequent tests until the mutation was detected (up to a maximum of 4 slides). Negative controls: For *KRAS* 3 Idylla™ KRAS Mutation Test (CE/IVD) cartridges were used with known *EGFR* positive samples as negative control, all three had a CQ value < 26 and none showed a *KRAS* mutation. For *EGFR* 3 Idylla™ EGFR Mutation Test (CE/IVD) cartridges were used with known *KRAS* positive samples as negative control, all three had a CQ value < 26 and none showed an *EGFR* mutation.

## Results

To assess the sample quality and DNA content of Idylla™ tested specimens, we used the total CQ value. In general, in our experiments (Tables 1, 2, and 3) samples with a sufficient TC% and with a total CQ value < 26, both *EGFR* and *KRAS* concordant mutations were detected. For that reason, we used a CQ value of 26 as the cutoff level for both *KRAS* and *EGFR*. For all negative *EGFR* and *KRAS* controls the CQ values remained below 26 and none showed a (false positive) mutation.

**Table 1:**
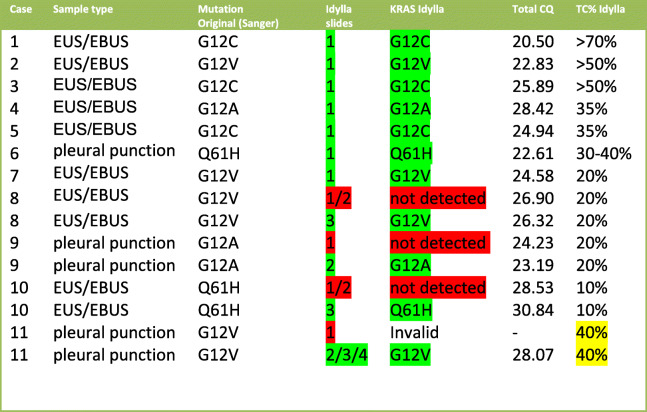
Idylla *KRAS. KRAS* mutation status using 1 to 4 slides of a total of 11 Sanger positive tested specimens were determined. Tumor cell % mentioned is the TC% estimated in the slides used for Idylla™. Because the samples were sequenced using Sanger sequencing the *KRAS* mutation VAF is unknown. Total CQ values were mentioned to assess the amplifiability of the DNA. For case 11 the CQ value of 2 slides is mentioned. For negative samples of cases 8 and 10, the lowest CQ total value detected is mentioned. Green indicates concordant results, red indicates non-concordant results (not detected or invalid). Yellow indicates that, although the tumor cell percentage was high, a possibility of a false-negative result was anticipated because of very low cell density (case 11)

**Table 2:**
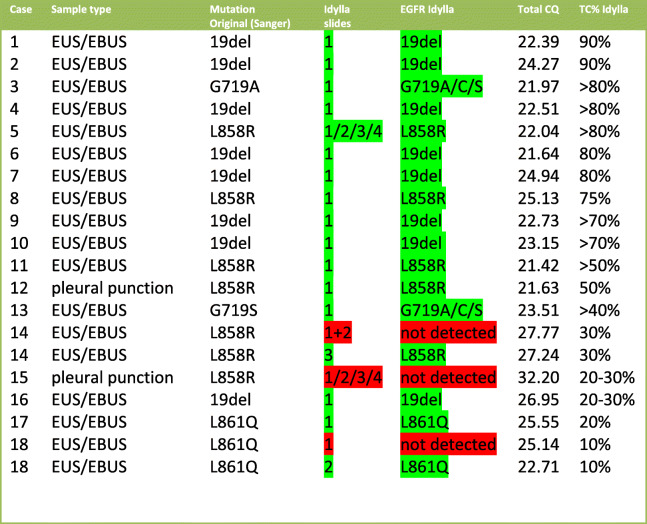
Idylla *EGFR* primary. *EGFR* mutation status was determined using 1 to 4 slides of a total of 18 Sanger-positive tested specimens. Tumor cell % mentioned is the TC% estimated in the slides used for Idylla™. Because the samples were sequenced using Sanger sequencing the *EGFR* mutation VAF is unknown. Total CQ values were mentioned to assess the amplifiability of the DNA. For case 5 the CQ value of 1 slide is mentioned. For both case 14 and 15 negative samples the lowest CQ total value detected is mentioned. Green indicates concordant result, red indicates non-concordant result (not detected)

**Table 3:**
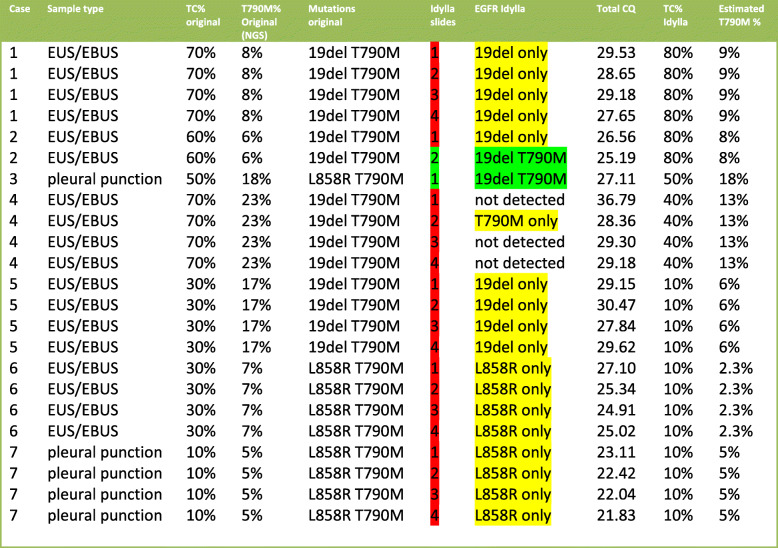
Idylla *EGFR* primary plus T790M**.** *EGFR* mutation status of 1 to 4 slides of a total of 7 T790M positive tested specimens (between 2,3 and 18% VAF), in addition to the primary *EGFR* mutation, were determined using Idylla™ *EGFR* cartridges. Total CQ values were mentioned to assess the amplifiability of the DNA. T790M VAF in Idylla™ samples was estimated using the formula (TC% Idylla™/TC% original sample)* T790M% original sample. Green indicates concordant result, red indicates non-concordant result (at least one of the expected mutations not detected). Yellow in the EGFR column indicates that only one of two expected mutations was detected

### Detection of KRAS mutations using Idylla™ cartridges

The comparative analysis of 11 *KRAS* positive cytological NSCLC FFPE Specimens using the Idylla™ KRAS Mutation Test revealed that all 11 cases were positive using Idylla™ and that the same genotype was found as with Sanger sequencing (Table 1).

**Fig. 1:**
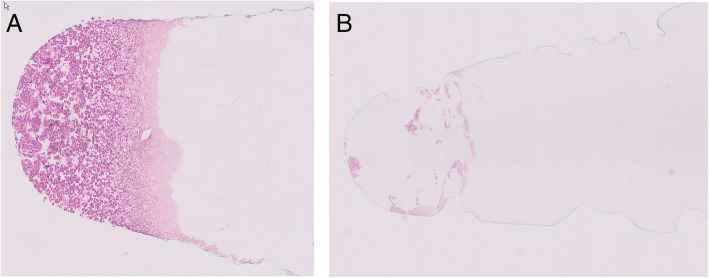
HE stained slides showing different cell densities in the cytological samples. **A** *EGFR* T790M case 1: normal/high cell density. **B** *KRAS* case 11; very low cell density

The first 10 *KRAS* mutated cases were initially analyzed with one 4 μm slide of material using Idylla™ *KRAS* cartridges and the number of slides was increased up to 3 if the KRAS mutation was not detected. Because the cell density in case 11 was extremely low (Fig. 1), we started with 4 slides for this case and lowered the number of slides in subsequent tests. In all samples, a concordant KRAS mutation was detected which gives a sensitivity of the KRAS mutation detection of 11/11*100% = 100%. Since none of the tests with negative controls showed a positive result, the specificity in this limited amount of samples was 100%.

### Detection of Primary EGFR mutations using Idylla™ EGFR cartridges

The comparative analysis of 18 *EGFR* positive cytological NSCLC FFPE Specimens using the Idylla™ *EGFR* Mutation Test revealed that 17 cases were also positive using Idylla™ and that the concordant genotype was found (Table 2.).

15 out of 18 cases could be analyzed with one slide only. All cases except case 15 showed the primary *EGFR* mutation. In case 15 the total CQ value of 32 shows that the amount of amplifiable DNA was low. A high CQ total means that the limit of detection (LOD) for the mutations will increase and hence also the risk for a potential false-negative result. In 17 of 18 cases, a concordant primary EGFR mutation was detected which gives a sensitivity of the primary EGFR mutation detection of 17/18*100% = 94%. Since none of the negative tests showed a positive result, the specificity in this limited amount of samples was 100%.

### Detection of Primary EGFR mutations plus EGFR T790M using Idylla™ cartridges

The analysis of 7 cytological NSCLC FFPE Specimens that previously showed both a primary *EGFR* mutation and a post-treatment *EGFR* T790M mutation, revealed that only 2 samples detected both the primary and the T790M *EGFR* Mutation (Table 3.).

The 7 cases had an estimated T790M VAF of 2,3 to 18% (see Materials and methods for estimation details). When the T790M mutation was not detected using 1 slide, more slides (up to 4) were used in subsequent tests until both primary and T790M mutation was detected. In 2 of 7 cases, the concordant T790M mutation/primary EGFR mutation combination was detected which gives a sensitivity of only 2/7*100% = 28% in our limited study.

### Invalid amplification curves of EGFR T790M

**Fig. 2:**
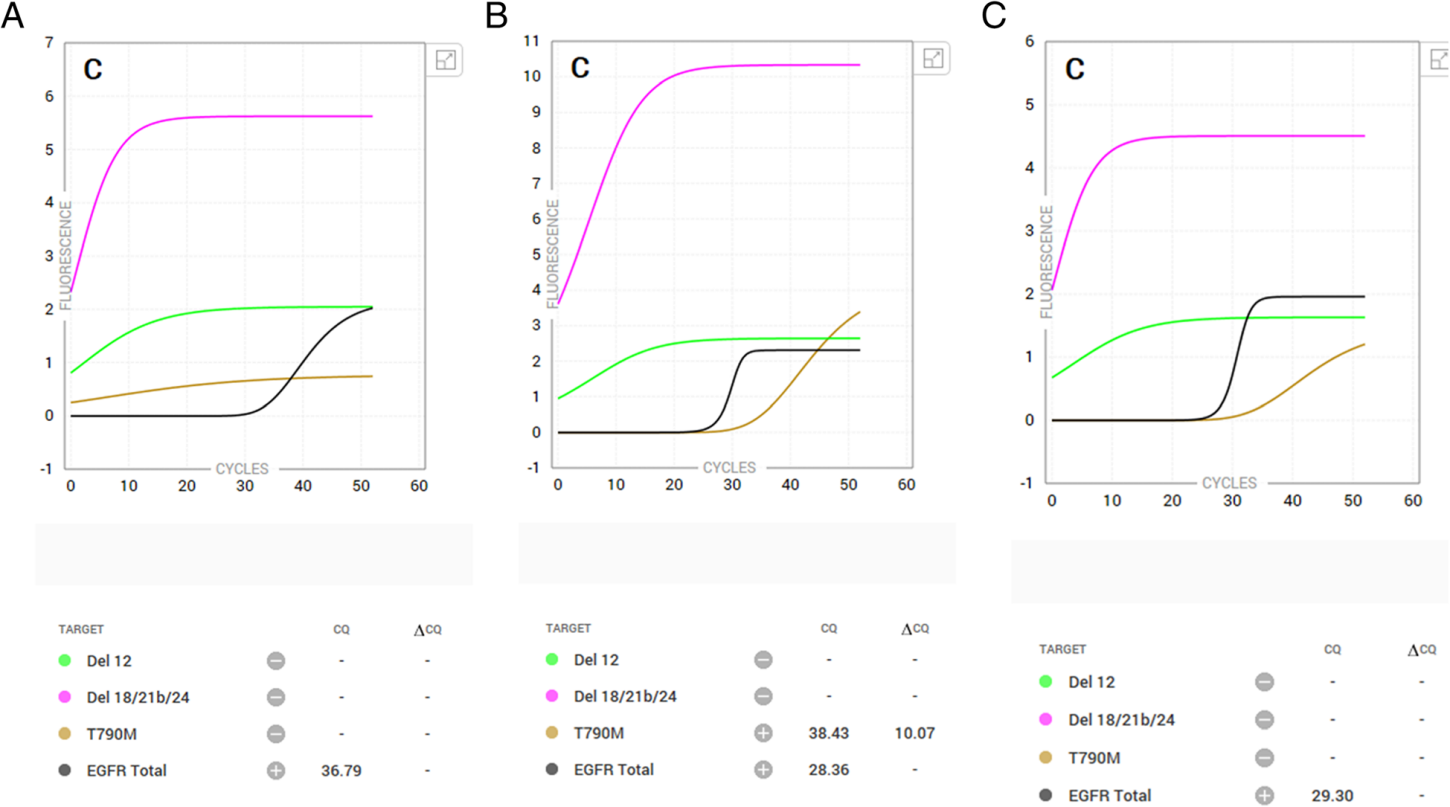
Amplification curves of *EGFR* T790M case 4. Idylla Explore analysis software amplification curves of **C** (which includes the curves containing the T790M mutation) are presented for analysis of EGFR T790M case 4 using **A** 1 slide, **B** 2 slides, C) 3 slides. Relevant curves: brown curve is the T790M-specific amplification curve. Black curve a sample processing control curve that reflects the amount of amplifiable DNA in the sample. Green (Del12) and pink (Del18/21b/24) curves are used for the detection of specific EGFR deletions, both deletion types are not present in this specimen

During the preparation of this manuscript Lee *et al* [13]. published a paper that, in agreement with our results, shows that the sensitivity of the Idylla EGFR test for the T790M mutation is low. They suggest that an invalid T790M amplification curve may indicate a possible false-negative result [13]. In this respect, one striking observation in our results was T790M case 4. This case had a negative result for T790M when 1, 3, or 4 slides of FFPE were used but had a positive result for T790M when 2 slides were used (Table 3.). Examination of the amplification curves (1,2 and 3 slides) showed that indeed T790M amplification curves of all three tests showed an increase, but that only the curve in the analysis of 2 slides reached a signal above the threshold (Fig. 2).

Although not the focus of this paper, as proof of principle, *BRAF* mutation status was tested using Idylla™ BRAF cartridges for two BRAF mutation-positive and three BRAF mutation-negative cytological FFPE cases and results were concordant with previous sequence data (data not shown).

## Discussion

Over the last years precision medicine has significantly improved patient outcomes for NSCLC adenocarcinoma and for an increasing amount of targets molecular testing is considered useful [15]. Although NGS can be used to analyze many genes at once and the DNA quantity required per gene analyzed is low, a threshold quantity of DNA is required for NGS that depends on the NGS panel and platform used [16]. A pathological diagnosis is often made on cytological FFPE specimens as a preferred method for minimally invasive diagnostics. In our experience, DNA extraction of cytological EUS/EBUS or pleural punction samples prepared as cytoblocks using AGAR and paraffin embedding results in very low DNA quantities, often too low for NGS testing. To ensure that the most important molecular markers are tested, quick and reliable testing with another method of hotspot regions of at least *EGFR* and *KRAS* (and preferably also BRAF) is needed for *NSCLC* adenocarcinomas to ensure optimal treatment possibilities with targeted therapy. This study aimed to evaluate the Idylla™ platform for mutation analysis in cytological FFPE samples of *NSCLC* patients. Cytological samples were prepared as cytoblocks using AGAR and paraffin embedding, resulting in FFPE material of a cytological sample. To assess the sample quality and DNA content of Idylla™ tested specimens, we used the total CQ value. Generally, in samples with a sufficient TC% and with a total CQ value < 26, both primary *EGFR* and *KRAS* mutations were detected concordant to the reference method. For that reason, we used a CQ value of 26 as the cutoff level for both *KRAS* and *EGFR*.

The concordant *KRAS* mutation was detected in all *KRAS* mutated specimens using the Idylla™ system. In all but one sample with a TC above 20%, the mutation was detected using a single slide (7 of 11). In 4 of 11 cases with a tumor cell percentage < 20% and in case 11 with high TC% but with very low cell density, the mutation was detected after increasing the number of slides analyzed. We, therefore, advise starting with 4 slides if the tumor cell percentage is 20% or lower or if cell density is very low. The average total CQ value gives an indication of the amount of amplifiable DNA in the sample and can be correlated with the analytical sensitivity. Therefore, if no mutation was detected (e.g. the negative controls) we considered the sample negative for a *KRAS* mutation if the sample has a total CQ value < 26. With a CQ total > 26 the probability of a false negative could not be ruled out and we considered, in this case, a negative sample unsuitable for evaluation (report as inadequate material). It must be noticed that the CQ cutoff level might be lab (pre-analytic phase) specific since other labs have determined other CQ cutoff values or even failed to set a cutoff value at all [13,14].

The primary EGFR mutation was detected in all *EGFR* mutation-positive samples with a TC above 30% (15 of 18 cases). In 2 *EGFR* positive cases with a TC below 30%, the EGFR mutation was detected after increasing the number of slides analyzed. We, therefore, advise starting with 4 slides if the TC is 30% or lower. In one *EGFR* positive sample (EGFR case 15) the EGFR mutation was not detected. This sample had a total CQ value of 32. Similar to the KRAS assay, if no mutation was detected we consider a sample negative for an *EGFR* mutation if the sample has a total CQ value < 26. With a CQ total > 26 the probability of a false negative cannot be ruled out and we consider the sample unsuitable for evaluation (report as inadequate material). According to these rules *EGFR* case 15 would have been rejected.

The EGFR T790M resistance mutation was detected alongside the primary mutation in only 2 of the 7 T790M positive cases. In one case (case 4) the EGFR T790M was found with 2 slides but not with either 1, 3, or 4 (unexplained, but note the high total CQ values; Table 3). In this sample, the primary mutation was not detected at all. The resistance mutation often occurs in only a small fraction of the tumor cells (in cases presented here in 2,3–18%), this makes detection more difficult. Because of the low percentage T790M mutant allele, more material is needed for the detection of T790M than for the primary EGFR mutation. Characteristically, EUS/EBUS and pleural punction cell yield typically are low after progression following treatment with first or second-generation *EGFR* TKI. This is reflected in the high total CQ values (compare Tables 2 and 3).

Personal communication with the company revealed that the Idylla™ EGFR Mutation test was designed for baseline testing and not for resistance testing. Because the EGFR T790M mutation comes with a high risk for deamination in FFPE samples with a high risk for false-positive results cutoffs were set rather high. Therefore, the Idylla™ EGFR-cartridge misses the T790M mutation in cytological samples with a low amount of amplifiable DNA and a low allelic fraction of the T790M mutant allele. Our observation that of the same EGFR T790M mutated sample 1, 3 and 4 slides did not produce a detectable EGFR T790M signal but 2 slides did (Fig. 2) indicates that the detection of EGFR T790M is indeed not reliable for ‘real world’ samples with poorer quality as Lee et al suggest [13]. Arcila et al. published a paper that also addresses the usefulness of Idylla for cytology samples [14]. In agreement with our results and the results of Lee *et al* [13], the authors conclude that the detection of the T790M mutation is less reliable than for primary EGFR mutations. They also notice that other relevant TKI-resistance mutations such as C797S and G724S are not detected by Idylla at all [14]. Taken together, our results combined suggest that the Idylla assay is not suitable for the assessment of cytological FFPE samples in the setting of TKI-resistance^13, 14.^

Cytological samples processed to cell blocks usually have a lower cell density than the tissue samples for which the Idylla™ tests have been developed (ref. 4 and Fig. 1). A sufficient quantity of amplifiable tumor DNA is required to be able to detect a mutation using Idylla™. However, because this method was developed to analyze a sample directly without prior DNA purification and quality analysis, other criteria to estimate whether a sample is suitable for Idylla™ were used. In general, the tumor cell percentage (TC%) of a specimen in which a mutation is present, is the most important factor whether or not a mutation is found using Idylla™ (Tables 1 and 2). In addition, it is also imperative that a sample has sufficient tumor cell quantity (= DNA content of the sample) to be able to detect a mutation. In Fig. 1 two extremes are pointed out: one sample with relatively high cell density but with a very low target (TC%) and a second sample with high (TC%) but very low cell quantity are shown. T790M case 1, high cell quantity (Fig. 1) and a TC 80% but with an estimated T790M VAF of only 9%, the T790M mutation was not detected with the Idylla™ system (Table 3.). On the other hand, the KRAS mutation was detected with only 2 slides of case 11 in a specimen with extremely low cell quantity (Fig. 1) but with an estimated TC of 40% (Table 1.). A high cell quantity does not always guarantee a sufficient amount of amplifiable DNA. The preparation and processing of the cell blocks may play a role in this. For either specimen with very low cell quantity (e.g. case 11 KRAS) or specimens with a low TC percentage, it is recommended to use multiple sections (e.g. 4) immediately.

In conclusion, the Idylla™ method is not suitable for cytological NSCLC adenocarcinoma samples prepared as cytoblocks using AGAR and paraffin embedding of patients with resistance to first and second-generation EGFR TKI’s. Primary *EGFR* and *KRAS* mutations could be detected reliably in our samples when TC percentage and the amount of amplifiable DNA (e.g. total CQ value) were taking into consideration. The Idylla™ *EGFR* and *KRAS* cartridges give very quick results that are easy to interpret with extremely low hands-on time. A big advantage of the method is that these mutations could be detected in specimens with a very low total-cell input, often already in only one slide, and also can generate reliable results in quantitatively low TC% samples. Results are obtained in samples with much less material than the amount of material needed for Next-generation sequencing. A disadvantage of Idylla™ tests is that they are single-gene tests and therefore only a few genes can be analyzed. The material used for Idylla™ is lost after analysis and cannot be used for other molecular tests. Together, this makes Idylla™a quick method, useful to analyze only the most important genes in primary NSCLC adenocarcinomas if the amount of material is insufficient to perform the complete set of molecular tests.

## Data Availability

All relevant data are included in the paper, no additional data available.
